# Comparing the Analgesic Efficacy of the Serratus-Intercostal Plane Block With Conventional Pharmacological Treatment in Patients Following Radical Mastectomy

**DOI:** 10.7759/cureus.91232

**Published:** 2025-08-29

**Authors:** Esther I De León-Herrera, Marco A Castellanos-López, Verónica Chávez-Macías, Juan F Hernández-Sierra, Quitzia L Torres-Salazar, Eloisa Rios

**Affiliations:** 1 Anesthesiology and Perioperative Medicine, Universidad Autónoma de San Luis Potosí, San Luis Potosí, MEX; 2 Surgery, Instituto Politécnico Nacional, Mexico City, MEX; 3 Anesthesiology, Instituto de Seguridad y Servicios Sociales de los Trabajadores del Estado, San Luis Potosí, MEX; 4 Anesthesiology, Universidad Autónoma de San Luis Potosí, San Luis Potosí, MEX; 5 Biomedical Sciences, Universidad Juárez del Estado de Durango, Durango, MEX; 6 Anesthesiology, El Instituto Mexicano del Seguro Social, San Luis Potosí, MEX

**Keywords:** mastectomy, opioid-sparing analgesia, postoperative pain, regional anesthesia, serratus-intercostal plane block

## Abstract

Introduction: Postoperative pain following radical mastectomy is common and can significantly impair recovery and quality of life. Regional anesthesia through the serratus-intercostal plane block (BRILMA) has been proposed as an effective strategy for pain control.

Objective: This study aims to compare the analgesic efficacy of BRILMA versus conventional pharmacological treatment in patients undergoing radical mastectomy.

Materials and methods: A prospective, observational cohort study with two comparative groups was conducted to evaluate the analgesic effectiveness of BRILMA versus conventional treatment following radical mastectomy. Women over 18 years scheduled for radical mastectomy were enrolled and assigned to groups based on the analgesic technique received during standard clinical care: the BRILMA group (ultrasound-guided block with 15 mL of 3% ropivacaine) and the control group (standard regimen with paracetamol, diclofenac, tramadol, dexamethasone, and ondansetron). The primary outcome was pain intensity, assessed using the Visual Analog Scale (VAS) at 4, 8, 12, and 24 hours postoperatively. Hemodynamic parameters were evaluated, and statistical analysis included Mann-Whitney U tests and chi-square tests.

Results: A total of 13 patients were included (six in the BRILMA group, seven in the control group). VAS scores were significantly lower in the BRILMA group at 8, 12, and 24 hours postoperatively (p<0.05). Additionally, lower systolic blood pressure was observed at 12 hours in the BRILMA group. No complications related to the technique were reported.

Conclusions: BRILMA is a safe and effective analgesic technique, demonstrating superiority over conventional treatment for controlling postoperative pain following radical mastectomy.

## Introduction

Breast cancer is the most common malignancy among women worldwide and constitutes a major public health concern in Mexico, where it ranks as the leading cause of cancer-related mortality in adult women [1]. It is estimated that 20% to 50% of patients undergoing radical mastectomy experience moderate to severe postoperative pain [2], which may progress to a chronic condition with a significant negative impact on quality of life.

Despite the use of conventional analgesic strategies that combine opioids, nonsteroidal anti-inflammatory drugs (NSAIDs), and adjuvants, a considerable number of patients experience inadequate pain control [3]. Recent evidence has encouraged the use of regional techniques as part of a multimodal approach due to their ability to provide effective analgesia, reduce opioid consumption, and enhance hemodynamic stability [4].

The serratus-intercostal plane block, known by the acronym BRILMA, stands for block of the lateral (cutaneous) branches of the intercostal nerves in the mid-axillary line, was first described by Diéguez García et al. in 2013 [5], and consists of the ultrasound-guided injection of a local anesthetic between the serratus anterior and intercostal muscles. This technique has proven to be technically simple, safe, and effective in blocking multiple intercostal nerves, making it a promising alternative for post-mastectomy analgesia [6].

Given the need for more effective strategies to manage postoperative pain in breast surgery, the aim of this prospective cohort study is to determine whether BRILMA provides superior analgesic efficacy compared to conventional treatment in women undergoing radical mastectomy.

## Materials and methods

A prospective, analytical, longitudinal cohort study was conducted to evaluate the effectiveness of the BRILMA block compared to conventional pharmacological therapy for postoperative pain management in patients undergoing radical mastectomy. The study was carried out in a tertiary referral center specializing in oncologic breast surgery and regional anesthesia. The study protocol was approved by the Institutional Research Committee (approval number: 003/2024) and the Institutional Ethics Committee (approval number: 001/2024) of the Instituto de Seguridad y Servicios Sociales de los Trabajadores del Estado, Hospital General de San Luis Potosí. This report adheres to the STROBE (Strengthening the Reporting of Observational Studies in Epidemiology) guidelines for cohort studies [7].

The study included women over 18 years of age, classified as American Society of Anesthesiologists (ASA) physical status I to III, who were scheduled for unilateral radical mastectomy for breast cancer and had provided written informed consent. Patients were excluded if they had known coagulopathies, allergies to local anesthetics, hemodynamic instability, active radiculopathies, or if they declined or withdrew consent at any stage of follow-up.

Participants were grouped based on the postoperative analgesia technique administered, as determined by routine clinical practice and attending anesthesiologist preference. In the BRILMA group, the block was performed at the end of surgery and prior to tracheal extubation. Patients were placed in the supine position with the ipsilateral arm abducted at 90°. A high-frequency linear transducer (6-13 MHz) was positioned longitudinally at the mid-axillary line at the level of the fifth intercostal space, and a 22-G, 70-90 mm needle was inserted in-plane in a caudal-to-cranial direction until reaching the fascial plane between the serratus anterior and the external intercostal muscles. A total of 15 mL of 0.3% ropivacaine was injected, with ultrasound confirmation of its spread across the fourth and fifth intercostal spaces. This technique provides analgesia by blocking the lateral cutaneous branches of the intercostal nerves, typically covering dermatomes T2-T6, which correspond to the anterolateral thoracic wall and axillary region [8]. The comparison group received conventional pharmacological analgesia with dexamethasone 4 mg, ondansetron 8 mg, paracetamol 10 mg/kg (maximum 1 g), diclofenac 1 mg/kg (maximum 75 mg), and tramadol 1 mg/kg (maximum 100 mg), as per institutional protocol.

The primary outcome was postoperative pain intensity, evaluated using the Visual Analog Scale (VAS) at 4, 8, 12, and 24 hours [9]. Secondary outcomes included heart rate, respiratory rate, and blood pressure (systolic and diastolic) measured at the same intervals. No serious adverse events related to the analgesic techniques were recorded.

Sample size was calculated using the formula for clinical trials comparing differences in means described by Velazco et al. [10], assuming a 95% confidence level and 90% statistical power to detect a minimum clinically significant difference of two points on the VAS, with an estimated standard deviation of 0.5 [11]. Based on this calculation, a total of 16 patients (eight per group) was deemed sufficient for preliminary analysis. Participants were allocated to each group through non-probabilistic sampling by consecutive case inclusion, according to the analgesic technique administered during standard clinical care.

Data were analyzed using IBM SPSS Statistics for Windows, Version 26 (Released 2020; IBM Corp., Armonk, New York, United States). The Kolmogorov-Smirnov test was used to assess the normality of continuous variables. Between-group comparisons for medians were conducted using the Mann-Whitney U test, and categorical variables were analyzed using the chi-square test. Intragroup changes were evaluated with the Wilcoxon signed-rank test. Analyses were performed per protocol, including only participants who completed all follow-up assessments.

## Results

A total of 16 patients were assessed between August and December 2024. Three were excluded: one due to hemodynamic instability detected during the preoperative assessment, which prevented surgery from being performed on the scheduled day, and two for receiving techniques not aligned with the study protocol. Thirteen patients were ultimately analyzed: six in the BRILMA group and seven in the control group.

No statistically significant differences were observed between groups in age, weight, height, body mass index (BMI), or ASA classification (p > 0.05). Clinical characteristics are summarized in Table 1.

**Table 1:** General characteristics of the study groups. Medians (q25-q75) are presented for continuous variables. The contrast between medians was performed with the Mann-Whitney U test.

Variable	BRILMA (n=6)	Control group (n=7)	p-value
Age (years)	64 (51-66)	59 (58-62)	0.534
Weight (kg)	68 (59-72)	70 (60-76)	0.628
Height (cm)	160 (156-165)	163 (150-170)	0.836
BMI (kg/m^2^)	24.8 (23-27)	26.6 (22-29)	0.628

**Figure 1:**
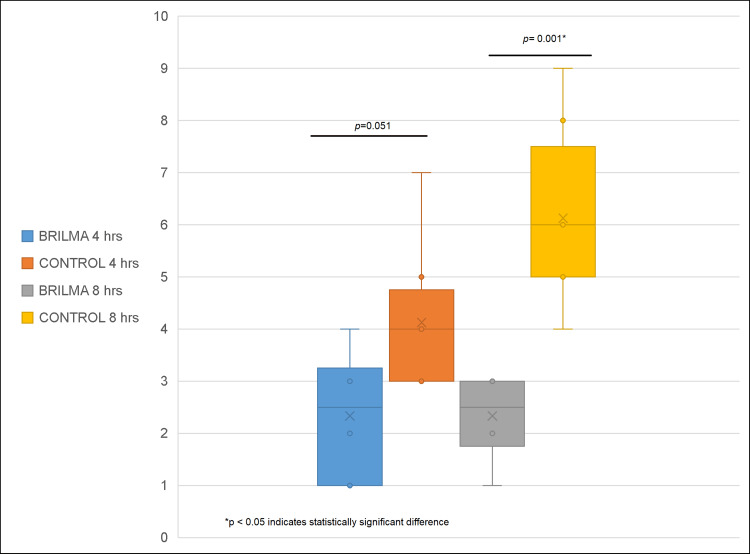
Pain intensity comparison at four and eight hours post-analgesia between BRILMA and control groups. VAS scores at four hours were 2.5 (q25-q75: 1-3.2) in the BRILMA group versus 4 (q25-q75: 3-5) in the control group, with no statistically significant difference (p=0.051). At eight hours, BRILMA demonstrated significantly lower pain scores, with a median of 2.5 (q25-q75: 1.75-3) compared to 6 (q25-q75: 5-8) in the control group (p=0.001). VAS: Visual Analog Scale

**Figure 2:**
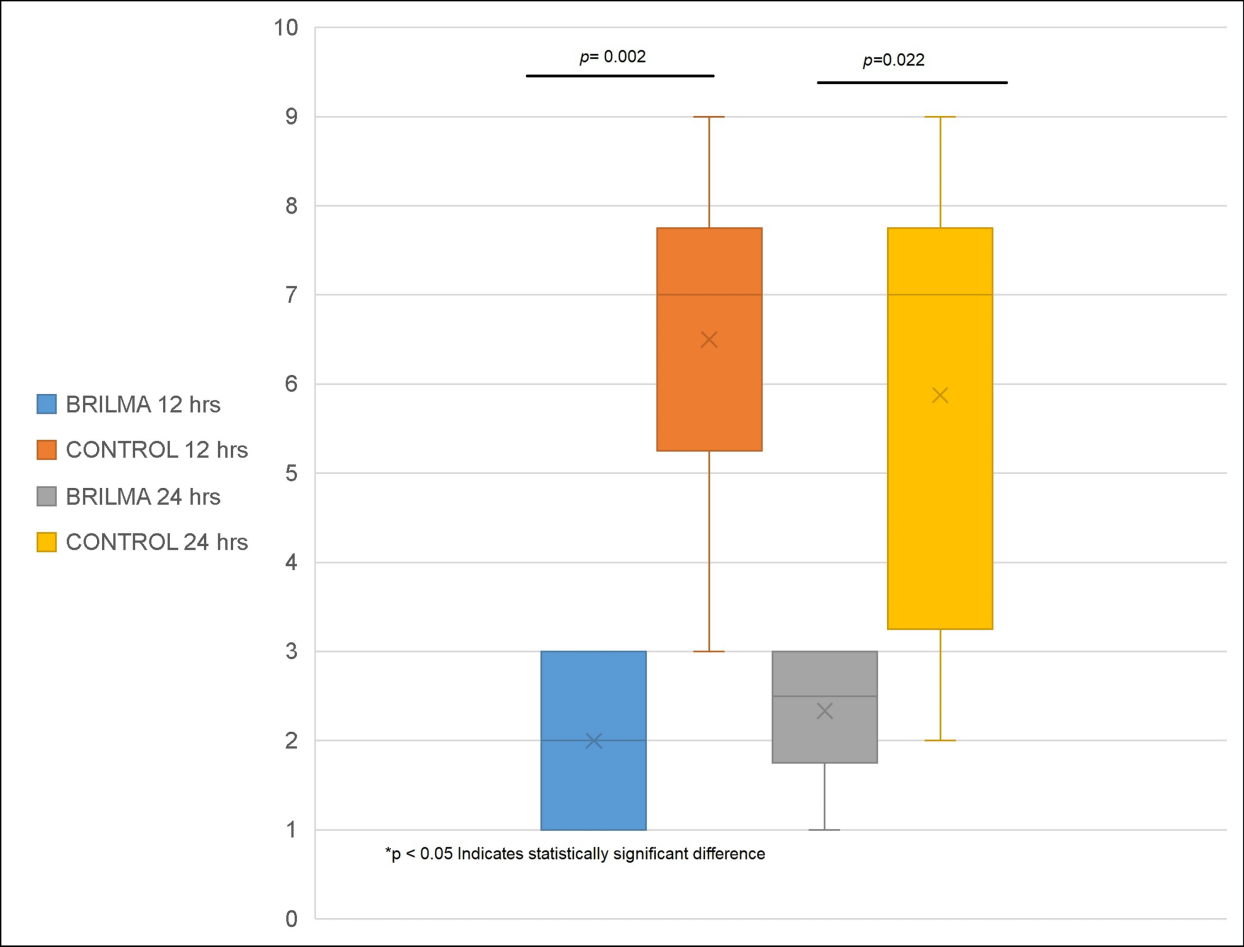
Pain intensity comparison at 12 and 24 hours post-analgesia between BRILMA and control groups. VAS scores at 12 hours were 2 (q25-q75: 1-3.2) in the BRILMA group versus 7 (q25-q75: 5-8) in the control group (p=0.002). At 24 hours, BRILMA patients reported a median of 2.5 (q25-q75: 1.75-3) compared to 7 (q25-q75: 3-8) in the control group (p=0.022), confirming a sustained analgesic effect. VAS: Visual Analog Scale

VAS scores were lower in the BRILMA group compared to the control group across all time points. At four hours, the BRILMA group showed a median of 2.5 (q25-q75: 1-3.2), while the control group had a median of 4 (q25-q75: 3-5), with no statistically significant difference (p=0.051). At eight hours, pain scores were significantly lower in the BRILMA group, with a median of 2.5 (q25-q75: 1.75-3) compared to 6 (q25-q75: 5-8) in the control group (p=0.001). At 12 hours, the BRILMA group reported a median of 2 (q25-q75: 1-3.2) versus 7 (q25-q75: 5-8) in the control group (p=0.002). At 24 hours, the BRILMA group maintained a median of 2.5 (q25-q75: 1.75-3), whereas the control group showed a median of 7 (q25-q75: 3-8) (p=0.022). These values are shown in Figures 1-2.

No significant differences were observed between groups in heart rate or respiratory rate at any time point. However, systolic blood pressure was significantly lower in the BRILMA group at 12 hours (p=0.022), with no differences in diastolic pressure. These values are detailed in Table 2.

**Table 2:** Hemodynamic and respiratory parameters identified in the study groups. Medians (q25-q75) are presented for continuous variables. The comparison between medians was performed using the Mann-Whitney U test. * Statistically significant difference (p<0.05).

Variable	BRILMA (n=6)	Control group (n=7)	p-value
Systolic pressure (mmHg) - 4 hrs	110 (98-132)	124 (110-137)	0.534
Systolic pressure (mmHg) - 8 hrs	101 (97-126)	130 (125-140)	0.628
Systolic pressure (mmHg) - 12 hrs	108 (99-121)	128 (120-150)	0.022*
Systolic pressure (mmHg) - 24 hrs	115 (107-126)	128 (115-130)	0.234
Diastolic pressure (mmHg) - 4 hrs	74 (60-90)	68 (68-78)	0.051
Diastolic pressure (mmHg) - 8 hrs	62 (58-85)	70 (65-80)	0.445
Diastolic pressure (mmHg) - 12 hrs	67 (63-77)	70 (68-73)	0.101
Diastolic pressure (mmHg) - 24 hrs	65 (60-78)	78 (70-82)	0.138
Heart rate (bpm) - 4 hrs	75 (64-89)	68 (62-77)	0.295
Heart rate (bpm) - 8 hrs	73 (63-88)	68 (65-75)	0.628
Heart rate (bpm) - 12 hrs	75 (64-88)	67 (62-75)	0.295
Heart rate (bpm) - 24 hrs	74 (65-83)	72 (65-82)	0.731
Respiratory rate (rpm) - 4 hrs	16 (15-16)	17 (16-18)	0.234
Respiratory rate (rpm) - 8 hrs	17 (14-18)	18 (18-20)	0.295
Respiratory rate (rpm) - 12 hrs	15 (14-18)	18 (16-20)	0.234
Respiratory rate (rpm) - 24 hrs	17 (14-18)	16 (16-19)	0.628

No major complications or adverse reactions related to the BRILMA technique were reported during the postoperative follow-up period.

## Discussion

The results of this prospective cohort study demonstrate that BRILMA provides superior postoperative analgesia compared to conventional treatment in patients undergoing radical mastectomy. Participants in the BRILMA group exhibited significantly lower VAS scores at 8, 12, and 24 hours postoperatively, indicating a sustained analgesic effect. These findings are consistent with those of Kumar et al., who reported that ultrasound-guided regional techniques, such as pectoral nerve (PECS) blocks, significantly reduced both pain intensity and opioid consumption within the first 24 hours after mastectomy [11].

A reduction in systolic blood pressure at 12 hours was also observed in the BRILMA group, potentially attributable to decreased sympathetic nervous system activation in response to nociceptive stimuli. This hemodynamic benefit aligns with the observations of Galán Gutiérrez et al., who noted that ultrasound-guided thoracic blocks not only improve analgesic comfort but also enhance cardiovascular stability in high-risk mastectomy patients [4].

One of the major strengths of this study is that it reinforces the evidence that interfascial plane blocks provide meaningful clinical benefits beyond statistical significance. In our cohort, BRILMA was not only effective in reducing pain scores but also showed a favorable safety profile, with no complications related to the technique. These observations mirror those reported in previous studies, where regional techniques consistently demonstrate an opioid-sparing effect and contribute to more stable hemodynamic parameters in the perioperative period. By describing the use of ultrasound guidance, validated pain scales, and structured follow-up, we aimed to present a pragmatic protocol that can be readily reproduced in similar oncologic surgery settings.

In contrast to the findings of Fernández Martín et al., who highlighted BRILMA’s effectiveness in reducing intraoperative fentanyl requirements during supraumbilical surgeries, our study did not assess intraoperative analgesia, limiting the feasibility of direct comparison. Nevertheless, the absence of complications in our cohort supports the safety profile of BRILMA, consistent with previous literature [6]. Sanllorente-Sebastián et al. similarly reported the successful application of BRILMA in an 87-year-old patient with multiple comorbidities, underscoring its utility in complex clinical scenarios [12].

The benefits observed in our population are clinically relevant. Adequate postoperative analgesia following mastectomy not only improves perioperative comfort but also reduces the risk of complications such as hypoventilation, secretion retention, or progression to chronic post-surgical pain. These results support the recommendations of major clinical guidelines, including those of the American Pain Society and the ASA, which advocate regional multimodal analgesia to optimize surgical outcomes [3].

Our findings are further supported by Alshawadfy and Al-Touny, who compared ultrasound-guided serratus anterior plane (SAP) block with PECS II block in modified radical mastectomy. Their randomized clinical trial demonstrated that SAP block significantly prolonged the time to first rescue analgesia and reduced total opioid consumption compared to PECS II, while maintaining hemodynamic stability and a low incidence of postoperative nausea and vomiting. Although our study did not directly compare BRILMA with PECS II or SAP blocks, the analgesic profile observed parallels the advantages of interfascial plane techniques described in their work, reinforcing BRILMA as a safe and effective option for thoracic wall analgesia [13].

Similarly, Sharma et al. conducted a randomized controlled trial comparing PECS block combined with general anesthesia versus general anesthesia alone in unilateral mastectomy. Their results demonstrated significantly lower intraoperative fentanyl use, delayed time to first rescue analgesia, and decreased postoperative opioid requirements in the PECS group. These findings parallel our observations regarding the opioid-sparing benefits of BRILMA, underscoring the role of ultrasound-guided regional techniques as a cornerstone of multimodal perioperative pain management in breast surgery [14].

Despite these advantages, it is important to acknowledge that the lack of double blinding in our study may introduce performance or expectancy bias. However, detection bias was minimized by ensuring blinded data analysis. Additionally, the small sample size limits the generalizability of our findings, although internal consistency and clinically relevant effect sizes support their validity.

Overall, BRILMA emerges as a safe and effective regional technique, well-suited for integration into multimodal analgesic protocols for radical mastectomy. Future randomized controlled trials with larger sample sizes and extended follow-up are warranted to further evaluate its role in preventing chronic postoperative pain and to assess its cost-effectiveness across diverse clinical settings.

## Conclusions

The BRILMA proved to be a safe and effective analgesic technique for controlling postoperative pain in patients undergoing radical mastectomy. Patients treated with BRILMA experienced significantly lower pain scores at 8, 12, and 24 hours postoperatively compared to those receiving conventional opioid and NSAID-based therapy. In addition to superior pain control, the BRILMA group demonstrated a trend toward greater hemodynamic stability, with a significant reduction in systolic blood pressure at 12 hours, without adverse effects attributable to the technique. These findings support the incorporation of BRILMA into multimodal analgesic strategies in oncologic breast surgery, particularly in settings aiming to reduce opioid use and enhance perioperative care quality.

However, given the limited sample size, the single-center setting, and the absence of double blinding, caution is warranted when extrapolating these findings to broader populations. Larger, multicenter trials with extended follow-up are required to validate these results and to further explore their implications for chronic pain prevention and long-term functional recovery.
